# A comparison of three methods to determine the subject matter in textual data

**DOI:** 10.3389/frma.2023.1104691

**Published:** 2023-06-02

**Authors:** George A. Barnett, Christopher Calabrese, Jeanette B. Ruiz

**Affiliations:** ^1^Department of Communication, University of California, Davis, Davis, CA, United States; ^2^Department of Communication, Clemson University, Clemson, SC, United States

**Keywords:** text analysis, topic modeling, community detection, cluster analysis, social media

## Abstract

This study compares three different methods commonly employed for the determination and interpretation of the subject matter of large corpuses of textual data. The methods reviewed are: (1) topic modeling, (2) community or group detection, and (3) cluster analysis of semantic networks. Two different datasets related to health topics were gathered from Twitter posts to compare the methods. The first dataset includes 16,138 original tweets concerning HIV pre-exposure prophylaxis (PrEP) from April 3, 2019 to April 3, 2020. The second dataset is comprised of 12,613 tweets about childhood vaccination from July 1, 2018 to October 15, 2018. Our findings suggest that the separate “topics” suggested by semantic networks (community detection) and/or cluster analysis (Ward's method) are more clearly identified than the topic modeling results. Topic modeling produced more subjects, but these tended to overlap. This study offers a better understanding of how results may vary based on method to determine subject matter chosen.

## 1. Introduction

The need to identify the diverse subject matter within a body of text has greatly increased due to the availability and access of online sources, including news and information websites, and social media. For example, what stories, events and issues are covered and how are they interpreted by different websites? What are the various topics discussed on social media? Three different methods are commonly employed to determine and interpret the subject matter of large corpuses of textual data; (1) topic modeling (Maier et al., 2018), (2) community or group detection (Danowski, 1993; Blondel et al., 2008), and (3) cluster analysis of semantic networks (Woelfel, 1997). This article compares the results of the application of these three different methods for the identification of the subject matter contained in large corpuses of textual data and their interpretability.

All three methods begin by determining the co-occurrence of words within the corpus. The frequency of the occurrence of bigrams (pairs of words or symbols) is determined by researchers' selection of the unit of analysis. This may be elements such as individual tweets (Calabrese et al., 2020; Featherstone et al., 2020), websites or online posts (Barnett and Hwang, 2006; Ruiz and Barnett, 2015; Jiang et al., 2018; Calabrese et al., 2019), emails (Danowski and Edison-Swift, 1985), grammatical units—sentences or paragraphs, open-ended survey responses (Rice and Danowski, 1993; Robbins et al., 2021), textual forms—manuscript titles (Doerfel and Barnett, 1999; Jiang and Barnett, 2018), abstracts (Barnett and Jiang, 2017), news stories (Barnett et al., 2013), letters (Jang et al., 1994; Fitzgerald and Doerfel, 2004), essays (Kwon et al., 2009) or speeches (Doerfel and Connaughton, 2009). Two words are said to co-occur if they are both present within the unit. Yang et al. (2022) propose the use of multiple units of analysis to increase the accuracy of the identification communities in semantic networks.

Alternatively, Danowski (1993) proposed the use of a sliding window of set length that moves through the text one or more words at a time. Two words are said to co-occur if they are both present within this window. Typically, the window is three or five words in width. The reason is that English has a subject-verb-object syntax. Hence, three words is the minimum window width. Since adjectives and adverbs are generally added to modify the noun or verb, a five-word window may be preferable. Although it may be as wide as seven to confirm to Miller (1956) notion that people can only process seven meaningful units of information, plus or minus two, at a time. This procedure has been widely adopted by other researchers (Woelfel and Stoyanoff, 1995; Carley et al., 2013; Diesner, 2014), and is especially useful when examining large bodies of text without an easily identified unit of analysis.

Generally, noise words such as prepositions, conjunctions, transitive verbs, and other meaningless symbols are removed prior to the creation of the list of bigrams (Doerfel and Barnett, 1999). Also, the words often undergo stemming, the combining of the forms of a word into a single word, its root term (Ji et al., 2022). For example, “compute”, “computing”, “computer”, and “computed” would be combined and treated as the symbol, “compute.” Once the noise words are removed, the words are stemmed, and the frequency of word co-occurrences are calculated, and one of the three methods examined in this research is applied to determine the text's different subjects. They are described below.

### 1.1. Topic modeling

Latent Dirichlet Allocation (LDA) (Blei et al., 2003; Maier et al., 2018), is widely employed to extract topics from text data (Ji et al., 2022). LDA uses an unsupervised probabilistic model that generates mixtures of latent topics from a corpus of text, where each topic is characterized by a distribution of words. This is determined by the text's unit of analysis, such as individual tweets or responses to open-ended survey questions. The words' frequency distributions are determined by the extent to which they co-occur in the analysis unit. The initial selection of topics is first sampled from a Dirichlet distribution, and a topic is chosen based on this distribution. LDA is a mathematical method that simultaneously estimates both the mixture of words that are associated with each topic and the assortment of topics that describes the content. The text is modeled as a number of subjects, with topics represented as distributions over words (Blei, 2012). To find these topics, LDA uses word co-occurrence patterns in the corpus, such that the more often two words co-occur in a document, the more likely they are assigned to the same topic (Aggarwal and Zhai, 2012).

An important issue for topic modeling is to determine the proper number of topics (k). If k is too small, the topics will be overly broad, while if it is too large, the topics may overlap. Models are selected and validated based on three criteria. One, selected models are based on the topic coherence score, which is a measure of a topic's semantic interpretability and association with well-defined semantic concepts (Newman et al., 2010). A high score denotes meaningful and interpretable topics. Two, models are validated based on human interpretation. Typically, one examines a sample of documents related to each topic and determines if the topics are easily interpretable. Third, intertopic distance mapping is also used to validate the topics. In an intertopic distance map, the distances between topics are calculated using Jensen-Shannon divergence, and then the intertopic distances are projected onto two dimensions (Sievert and Shirley, 2014; Mabey, 2018). If they overlap, the semantic contents of the topics are similar. Based on interpretation and intertopic distance map, one selects the model in which the topics are independent and interpretable.

### 1.2. Community detection

The community detection method was developed to identify subgroups in networked systems including social and biological networks (Girvan and Newman, 2002). Community detection focuses on the property in which the network nodes are joined together in tightly knit groups, and between which there are only looser connections. The method for detecting such communities or groups uses betweeness centrality to find community boundaries. By removing those nodes that have the highest centrality, subgroups or communities are revealed. For semantic networks, the co-occurrences of words are treated as a valued sociomatrix, **W**, where w_ij_ is the frequency word *i* and word *j* occur in the text. Words in the same community (group) are more densely connected with each other than with others in the network of text as described by the frequency of word co-occurrences. The identification of highly interconnected symbols reveals the subject of each linked group of words. Similarly, Danowski (1993) identified groups of words based on their proximate co-occurrences where members of groups shared more than 50% of their links with one another. The groups reveal different subjects, and the centrality of words within each community or group facilitates the interpretation of the subject. Nodes that had connections to multiple groups and other intergroup linkers are what the communication science literature labels “liaisons” (Rogers and Kincaid, 1981; Rice and Danowski, 1993). Liaison words are the most central concepts. Yuan et al. (2013) and Danowski et al. (2021) used Clauset-Newman-Moore community detection and examined the most central words in each to interpret and label the subjects.

Blondel et al. (2008) have proposed an algorithm to detect communities based on modularity optimization. Modularity is a community detection statistic that identifies different groups by determining the fraction of the links that fall within a given group (Newman, 2004) and is commonly used in the study of semantic networks (Jiang and Barnett, 2018; Jiang et al., 2018; Featherstone et al., 2020; Robbins et al., 2021). A threshold value of 0.4 (40% of the edges are within a given community) or above should be obtained for meaningful community identification (Blondel et al., 2008), that is somewhat less than a majority of nodes are tied exclusively to only a subset that excludes others in the network.

### 1.3. Cluster analysis

Community detection and cluster analysis are often used interchangeably in the literature although community detection focuses on network structure as a function of connectivity. The method creates separate groups by removing the nodes (words) with the highest betweenness centrality. Clustering focuses on a single modality, specifically, node attributes (the frequency of word co-occurrence) to group network objects (Inuwa-Dutse et al., 2021). Hierarchical cluster analysis (Girvan and Newman, 2002) is the traditional method for detecting groups in networks. Hierarchical cluster analysis has been used frequently to determine the different subjects in large bodies of text (Doerfel and Barnett, 1999). It treats the frequency of word co-occurrence matrix (**W)** as a covariance matrix, with the value of w_ij_, representing how closely related the words are. Cluster analysis's goal is to maximize within-group subject homogeneity and between-groups heterogeneity, to determine the exclusivity of the cluster's subject matter. Procedurally, one takes the individual words (w_i_) in the matrix and merges them together one at a time in a series of sequential steps in order of their co-occurrence. As words are added (from greatest frequency to least), a tree graph or dendrogram is created showing a nested set of increasingly large clusters (connected subsets of words), which identify exclusive groups. These clusters, which at the lowest level has two words connected, demonstrate the strength of their relationship. The selection of the level which determines group membership is arbitrary and depends on the researcher's interpretation.

Because the individual words (i) in the text have varied co-occurrences with the others (j, k,…) in a semantic network, there are many different criteria in hierarchical cluster analysis that can be used when adding a word to a cluster and/or combining two clusters (Yim and Ramdeen, 2015). The *single linkage* or the minimum method, defines the relationship between two clusters as the minimum co-occurrence between one word to a cluster or the minimum from the first cluster and one word in a second. This method often leads to chains among the clusters. The *complete linkage* or maximum method is similar to the single linkage measure, but instead of using the minimum co-occurrence between pairs of words, it considers the maximum between pairs of words or clusters. It often leads to close clusters not being merged. Because of the limitations of both the single and complete methods, there is the *average linkage* method, which takes an average of the co-occurrences between clusters. Also, there is the *Ward's method* in which the linkage function between two clusters is computed as the increase in the error sum-of-squares after merging two clusters (Ward Jr, 1963). Further, there are non-hierarchical forms of cluster analysis that may be applied to semantic networks (Morissette and Chartier, 2013). One such method is, such as *k-means* clustering. However, it requires specifying the number of clusters prior to the analysis, which represents a severe drawback when examining large corpuses of text (Yim and Ramdeen, 2015). Woelfel (1997) advocates the use of non-hierarchical cluster analysis for the analysis of neural networks when applied to text data.

The goal of this research is to compare the three different methods (topic modeling, community or group detection and cluster analysis of semantic networks) used to determine the subject matter of textual data. This raises the following research questions. Do the three methods produce different topics from textual data? Do the three methods identify the same topics? Does one method produce results that are more easily interpreted than the others?

## 2. Materials and methods

### 2.1. Datasets

Two different data sets were used to compare the three different methods used for the determination and interpretation of the subject matter of large corpuses of textual data. The first dataset examined discussions on Twitter surrounding HIV pre-exposure prophylaxis (PrEP) (Calabrese et al., 2022) and consisted of 16,138 original tweets (from April 3, 2019 to April 3, 2020). Tweets were extracted using the R package *twint* (Zacharias, 2020) with the search terms “pre-exposure prophylaxis,” Truvada,” “Descovy,” and the hashtag, “#PrEP.”

The second dataset examined Twitter content about childhood vaccinations and included 12,613 tweets (from July 1, 2018 to October 15, 2018) (Featherstone et al., 2020). The entire archive of English language tweets was collected from Twitter's Premium API using Boolean search methods with keywords “vaccine,” “vaccination,” “vax,” “shot,” “immunization,” and “immunization,” in combination with childhood vaccine types “MMR,” “Tdap,” and “HPV.”

### 2.2. Analysis procedures

First, the datasets were uploaded to *R* (Version 4.0.3) where we ran data cleaning steps to remove noise, such as symbols, hyperlinks, punctuation, and whitespace. Then, syntactically functional words (articles, conjunctions, prepositions) were removed and different forms of the common words (e.g., signify and signifies) were stemmed. After data cleaning, we then created the corpus of words using the text mining *tm* package (Feinerer, 2013).

For topic modeling, we ran *R* to create our document term matrix, which is used for both estimating the *k* number of topics and for the topic modeling itself. We then used the *stm* package (Roberts et al., 2019) to fit a set of models with different *k* number of topics from a range of 5 to 12 topics. This allows for an examination of their resulting diagnostic plots, which include coherence scores, residual scores, held-out likelihood, and lower bound. We also examined the intertopic distance maps to examine whether the topics were independent and distinguishable using the *LDAvis* package (Sievert and Shirley, 2014). We then used the *topicmodels* (Grün and Hornik, 2011) package to fit the topic models. *Topicmodels* was selected because it builds upon the *tm* package results. This package uses a *Gibbs* sampling algorithm, which can be advantageous over the variational expectation-maximization algorithm (VEM) algorithm (Blei et al., 2003) because it requires less memory to process large amounts of data. We then fit the topic models with our determined *k* topics and examined the primary words within each topic.

For the community detection analyses, we converted the cleaned data into text files to be processed in *ConText* (Diesner, 2014). In *ConText* (Diesner, 2014), the semantic network was generated using the edited texts based on word co-occurrence. The basic network data set is an *n* x *n* matrix **W**, where *n* equals the number of nodes (words) in the analysis and w_ij_ is the measured relationship between nodes i and j with the word serving as the unit of analysis based on the frequencies of the word occurrences. Links were created for words that occurred within five words of one another within each tweet. This procedure is equivalent to tokenizing the text. That is splitting the body of text into smaller units that can be more easily assigned meaning. The semantic network was created using the network visualization software, *Gephi* (Bastian et al., 2009; Jacomy et al., 2014). The ForceAtlas2 algorithm was used to visualize the network. This open-source, user-friendly software is widely used by the network science community because it has been shown to be comparable in speed and quality to other layouts. The size of the word label indicated how frequently the word occurred. The thickness of each link represented the number of co-occurrences between two words. The more closely related the words were, the shorter the link distance. Community detection was also conducted using *Gephi* through the modularity calculation (Blondel et al., 2008). In the graphic display of the semantic networks, the distinct subjects are portrayed using different colors. The modularity calculation algorithm is advantageous over other community detection methods because it takes less time to compute and can determine communities within larger datasets.

For cluster analysis of the datasets, we ran *R* and transposed the document term matrix and then created the distance matrix using Euclidean distance measure. Then, we ran the hierarchical cluster analysis on the distance matrix using Ward's method (Ward Jr, 1963) and plotted the resulting dendrograms. To estimate the ideal number of clusters, we calculated the *Gap* statistic, which has shown to perform better than other cluster number determination measures (Tibshirani et al., 2001). The *Gap* statistic uses the output of a clustering algorithm by comparing the change in within-cluster dispersion with what would be expected under an appropriate reference null distribution.

Lastly, to compare each text analysis method, we examined the top ten most frequent words in each dataset. We compared the similarities and differences between the resulting topics, communities, and clusters for each of the top ten words.

## 3. Results

### 3.1. Topic modeling

**Figure 1:**
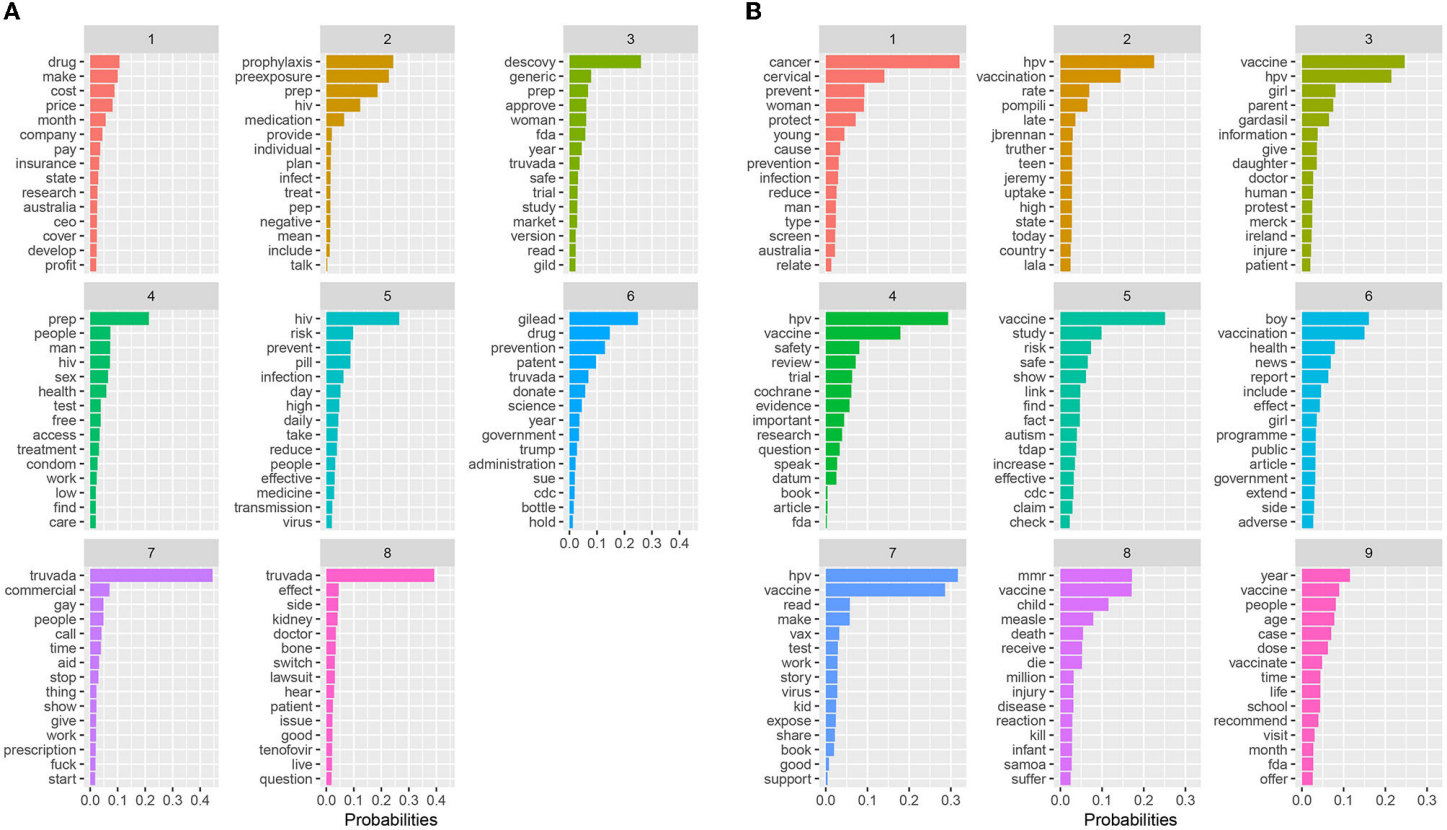
Topic modeling results for **(A)** PrEP and **(B)** Childhood vaccination.

**Figure 2:**
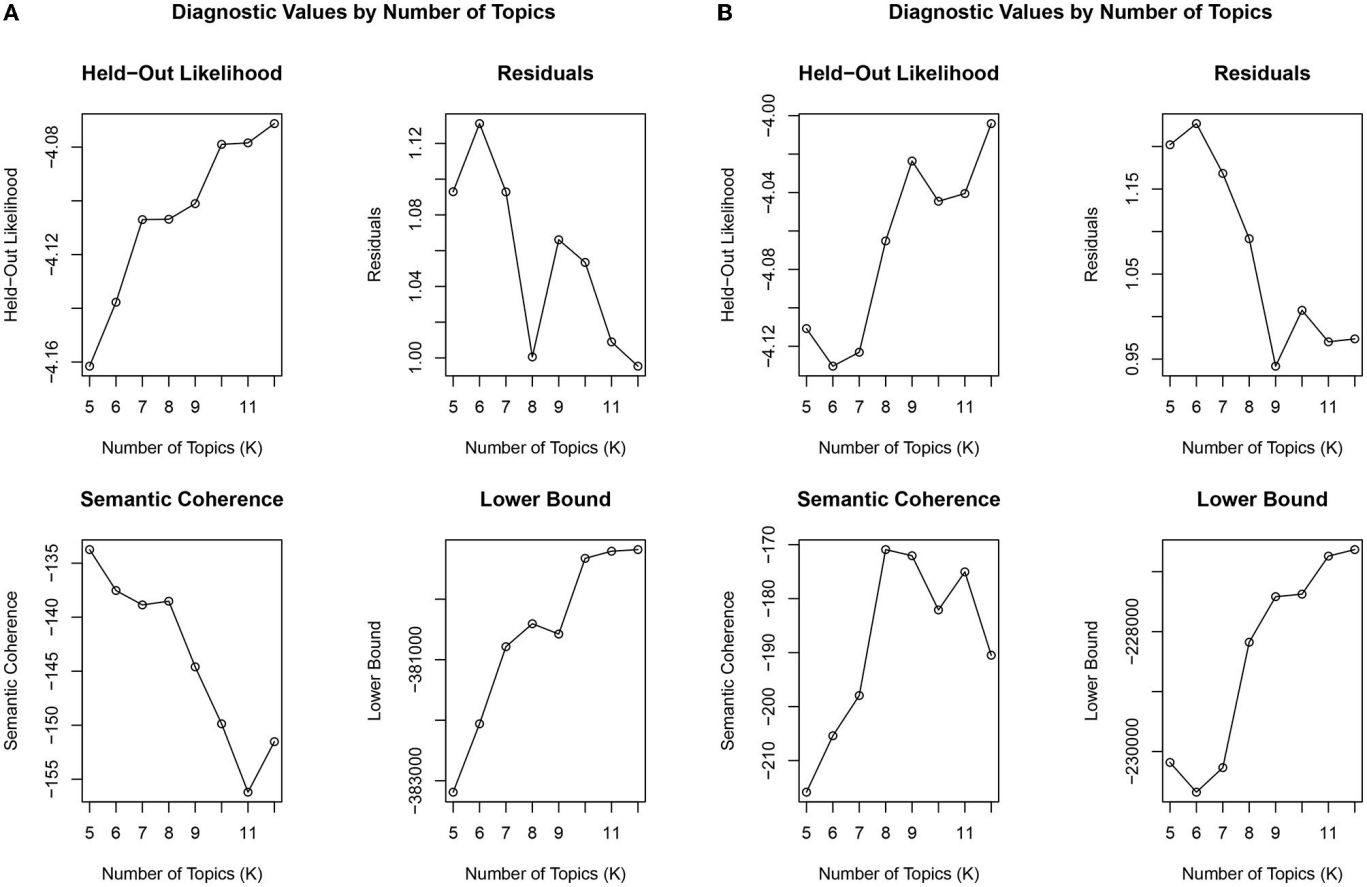
Diagnostic values for the number of topics for **(A)** PrEP and **(B)** Childhood vaccination.

Based on our topic modeling analysis, eight topics emerged from the PrEP dataset (see Figure 1A). Eight topics resulted in the highest coherence with the lowest residuals (Figure 2A). Topic 1 primarily focused on the costs of PrEP in comparison to the cost of manufacturing the medication. For example, the comparison between the cost of PrEP in the U.S. vs. Australia is depicted. Topic 2 describes PrEP and PEP (post-exposure prophylaxis) as medications to take for preventing HIV infection. Topic 3 focused on alternative medications for PrEP, including Descovy (recently FDA approved) and a potential generic version of PrEP. The word *woman* was also in this topic, potentially indicating that Descovy is not yet approved for individuals assigned female at birth. Topic 4 depicts preventive methods that should be taken while on PrEP. This includes finding and accessing healthcare, regularly testing for sexually transmitted diseases, and regular condom use. Topic 5 focused on how PrEP is an effective prevention method for reducing one's risk for HIV transmission by taking the medication daily. Topic 6 focuses on the legal issues related to PrEP. This involved the multiyear agreement with the Trump administration to donate PrEP and PrEP patent disputes between Gilead and the U.S. government. Topic 7 described opinions about the Truvada commercials, including some positive and negative opinions surrounding its focus on gay individuals. The last topic (Topic 8) focused on patients switching from Truvada to Descovy. It included details on Truvada's potential side effects of kidney damage and bone density loss.

**Figure 3:**
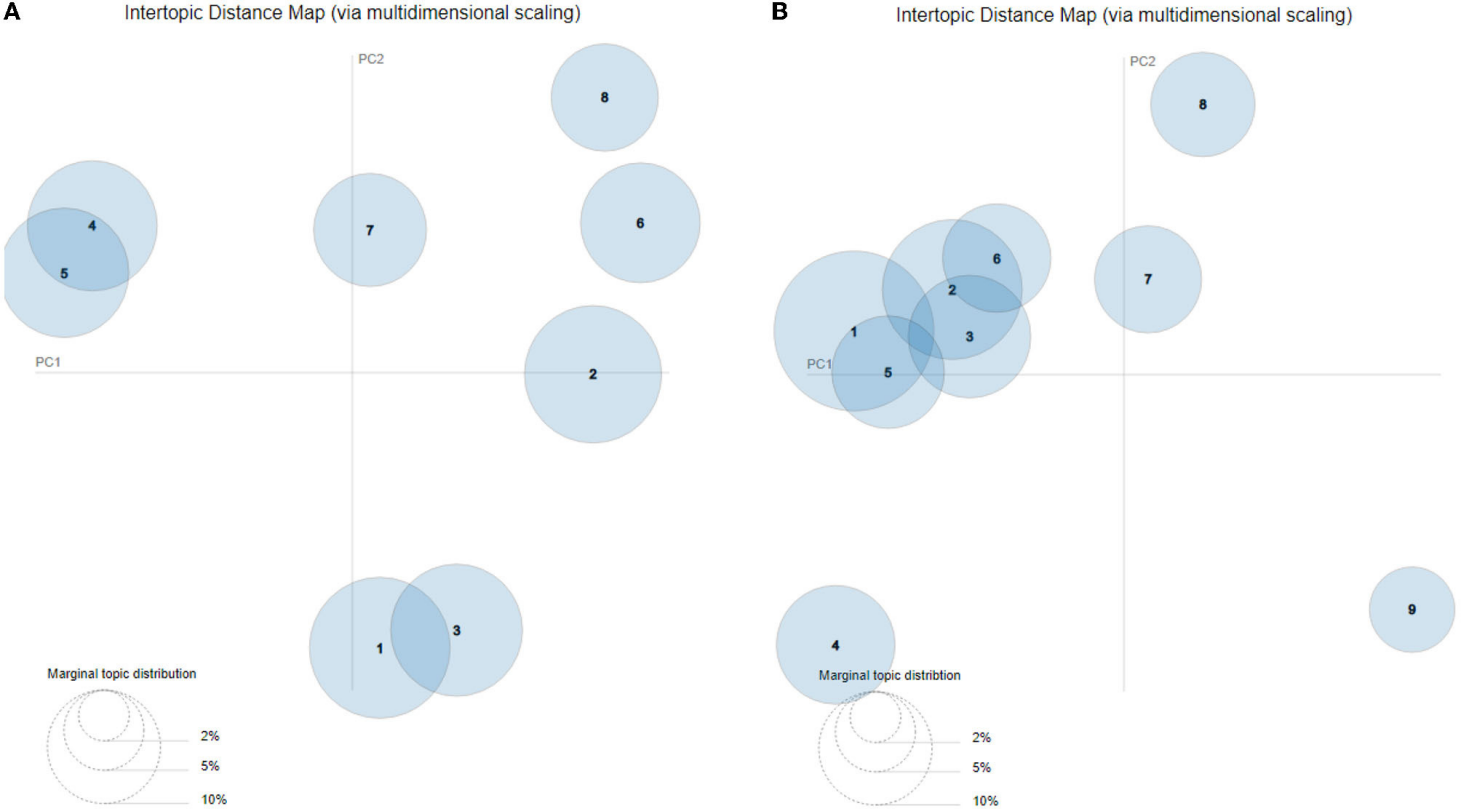
Intertopic distance map results for **(A)** PrEP and **(B)** Childhood vaccination.

While coherence and the other diagnostic plots suggested eight topics in the PrEP data (Figure 2A), the intertopic distance map (Figure 3A) indicated that some of the topics overlapped. Topics 1 and 3 both dealt with pharmaceuticals, their costs and alternatives. Topics 4 and 5 were concerned with HIV prevention. Figure 3A shows this lack of exclusivity and suggests that there are six, rather than eight topics as previously described.

Nine topics emerged from our childhood vaccine dataset (see Figure 1B). Figure 2 shows the nine topics that scored a higher coherence and produced the lowest residuals when compared to the other topics. Topic 1 focused on cervical cancer prevention and protecting youth against infection. Topic 2 describes the issue of vaccines internationally. Topic 3 focused on providing information about the HPV vaccine (Gardasil) to parents and their daughters. This topic included the protests against the vaccine in Ireland. Topic 4 focused on the Cochrane HPV vaccine review. Topic 5 focused on the debunked myth that the vaccines cause autism. Topic 6 focused on the inclusion of boys in vaccination programs, as well as discussions of side and adverse vaccine effects. Topic 7 depicts reading about the HPV vaccine. Topic 8 details the MMR vaccine and deaths related to measles, including the measles outbreak in Samoa. And the last topic (Topic 9) focuses on recommended vaccine schedules for school and over the lifetime.

As is the case with the PrEP data, the intertopic distance map (Figure 3B) of the childhood vaccine text suggested fewer exclusive topics than the nine suggested above. As illustrated in Figure 3B, only five exclusive topics emerged. There is extensive overlap among Topics 1, 2, 3, 5, and 6.

### 3.2. Community detection

**Figure 4:**
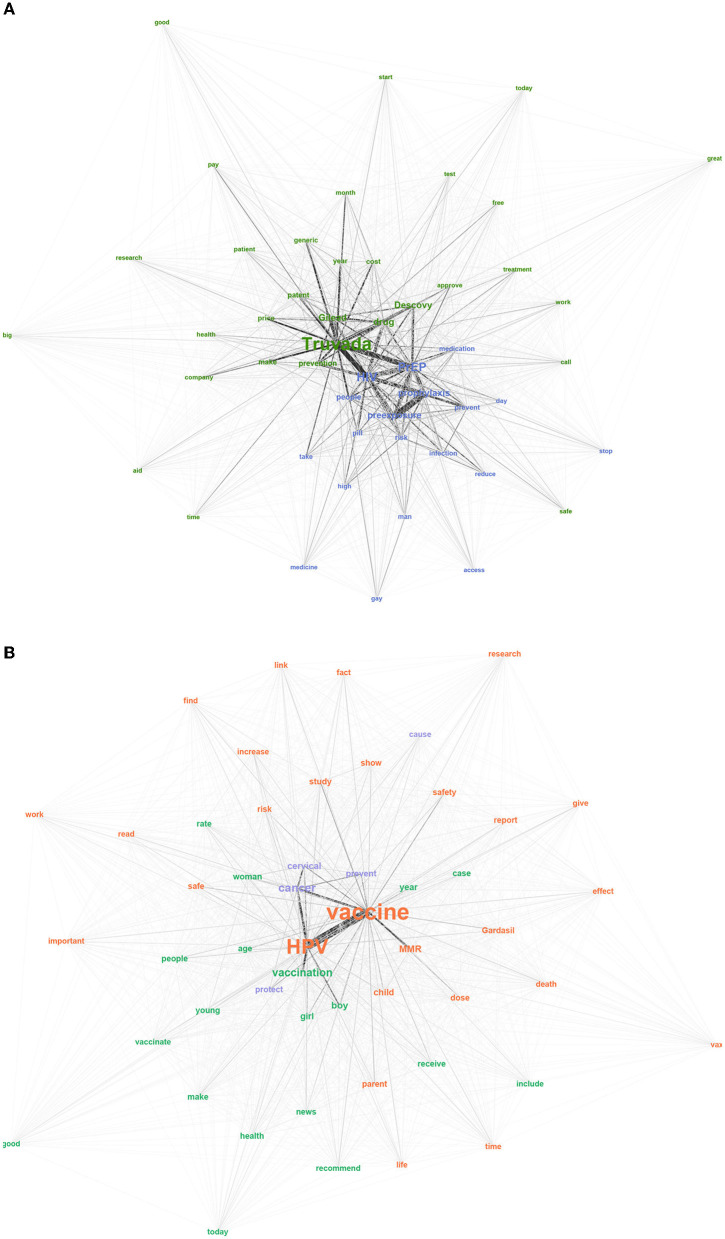
Semantic networks with communities on **(A)** PrEP and **(B)** Childhood vaccination.

Figure 4 presents the semantic networks where each color represents an identified community. Two communities with extensive connection between them are depicted in the PrEP dataset (Figure 4A). The modularity was very low, 0.151, indicating that the two communities are not independent of one another. The largest community is in purple and consisted of 79.6% of the total network, while the smaller community consisted of 20.4% of the network. The purple community depicts the general aspects of PrEP, including what the medication does and how to take the medication. For example, the most central words involve *PrEP, HIV*, and *preexposure*. All of these relate to the name of the medication, while words such as *prevent, risk, pill, day*, and *reduce* indicate the purpose and use of PrEP. The green community centers on the brand names of the medication, as well as the barriers related to obtaining the medication. For example, the brand names *Truvada* and *Descovy* are central to the network, as well as *Gilead*, the company that owns the patent. Other words in this community such as *price, cost*, and *patent* indicate issues related to access barriers. Noticeably, *approve* is closely tied to *Descovy*, since *Descovy* was recently approved for certain populations.

For the childhood vaccination dataset, three communities emerged (Figure 4B). As was the case with the PrEP data, the modularity was below the 0.4 threshold, 0.059. The displayed communities have many connections and are not independent. The largest community is in purple and represented 66.9% of the network. The orange community consisted of 19.4% and the green community consisted of 13.7% of the network. The purple community focuses on one main theme - that the HPV vaccine can prevent and protect against cervical cancer (*cancer, cervical, prevent, protect*). Within the orange community, most of the words focus on the HPV vaccine including its safety and potential risks (*HPV, vaccine, Gardasil, safety, death*). While the community primarily focuses on the HPV vaccine, the *MMR* vaccine was also included in this community. The last community in green highlights vaccine recommendations, such as *vaccination, boy, girl, recommend*, and *young*. These words may indicate that the focus of discussions on Twitter are related to informing or promoting childhood vaccines, especially the HPV vaccine for preteen girls and boys.

### 3.3. Cluster analysis

**Figure 5:**
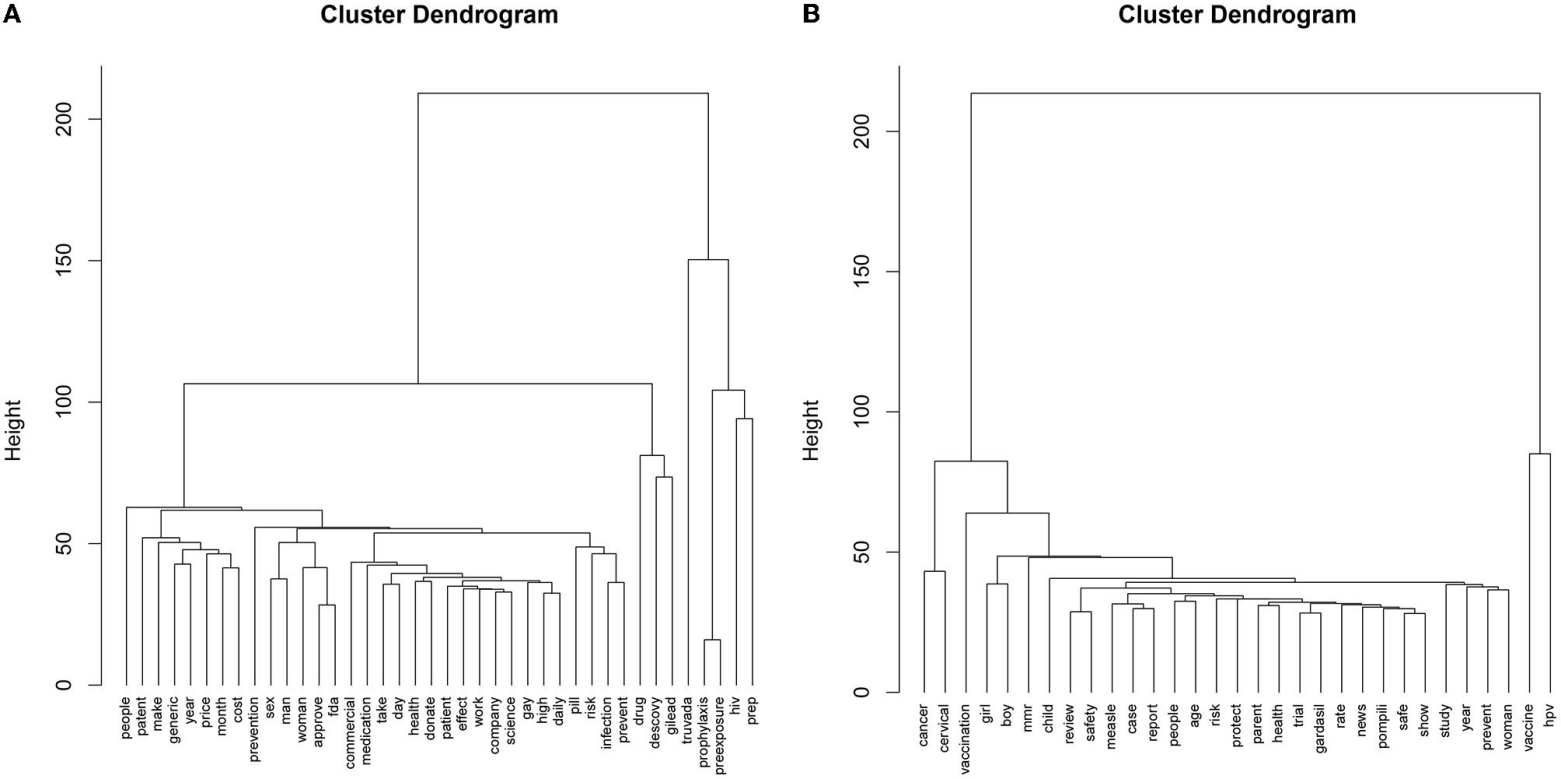
Cluster dendrograms for **(A)** PrEP and **(B)** Childhood vaccination.

Figure 5 depicts the dendrograms for each dataset. For the PrEP dataset (Figure 5A), the *Gap* statistic method determined six clusters as the optimal number. Due to the large differences in frequency between words, many individual or few words filled their own clusters. Cluster 2 includes *drug, Descovy*, and *Gilead*, while others consisted of *Truvada* (Cluster 3), *prophylaxis* (Cluster 4), *HIV* (Cluster 5), and *PrEP* (Cluster 6). The first cluster concentrates on barriers to access (*access, cost*, and *requirements)*, who should take PrEP for preventing the risk of infection, and how to take the medication (Cluster 1). Despite the large differences between clusters, each level within is interpretable.

For the childhood vaccine dataset (Figure 5B), we determined that the optimal number of clusters is two based on the *Gap* statistic. One cluster consists of the words *HPV* and *vaccine*. The other cluster describes the function of the HPV and MMR vaccines, their safety, and related news and reports.

### 3.4. Comparison of text analysis methods

To add an additional layer in comparing the three text analysis methods used in this study, we examined and compared the top ten most frequent words in each dataset. Table 1 displays the most frequent words and their associated topics, communities, and clusters. In general, words were grouped into similar categories for each method. For example, in the PrEP dataset, *HIV* and *PrEP* both emerged in topics 2 and 4 from the topic modeling analysis and are both within the purple-labeled group in the community detection analysis. Although the two words were in their own distinct clusters, they were closely linked in the dendrogram. Because of the detail in the topic modeling results, there may be an overlap in topics, despite resulting in different communities and clusters. For example, *Truvada* (Topics 3, 6, 7, and 8) and *people* (Topics 4, 5, and 7) overlap on Topic 7, but differ in their community and cluster. Because both words are also present in other topics, the community detection and cluster analyses found them to be grouped separately.

**Table 1:** Comparison of text analysis methods.

**Term**	**Frequency**	**Topic(s)**	**Community detection**	**Cluster analysis**
**PrEP dataset**				
Truvada	10857	3, 6, 7, 8	Green	3
HIV	5623	2, 4, 5	Purple	5
PrEP	5584	2, 3, 4	Purple	6
Drug	3275	1, 6	Green	2
Gilead	3045	7	Green	2
Descovy	3022	3	Green	2
Preexposure	2998	2	Purple	4
Prophylaxis	2819	2	Purple	4
People	1794	4, 5, 7	Purple	1
Prevention	1581	6	Green	1
**Childhood vaccine dataset**				
Vaccine	7800	3, 4, 5, 7, 8, 9	Orange	1
HPV	6679	2, 3, 4, 7	Orange	1
Cancer	2146	1	Purple	2
Vaccination	1852	2, 6	Green	2
MMR	1092	8	Orange	2
Boy	1029	6	Green	2
Cervical	941	1	Purple	2
Girl	745	3, 6	Green	2
Child	738	8	Orange	2
Year	728	9	Green	2

In the childhood vaccine dataset, we find similar results that demonstrate the similarities between text analysis methods. For example, *vaccine* and *HPV* share three topics (Topics 3, 4, and 7) based on our topic modeling analysis. These are also in the same community (orange) based on our community detection analysis and in the same cluster based on our cluster analysis. Similarly, words such as *vaccination, boy*, and *girl*, were all found in Topic 6, in the same community (green), and within the second cluster. In addition, *cervical* and *cancer* shared Topic 1, resulted in the same community (purple), and were in the second cluster. Similar to the PrEP dataset, groupings of words in the vaccine dataset tended to differ slightly with the topic modeling method. This was mainly due to the higher number of topics that resulted from topic modeling.

## 4. Discussion

This study examined three different text analysis methods, including topic modeling, community detection, and cluster analysis using two unique health-related datasets to compare each method's strengths and weaknesses in identifying the main subject matter within the text. We find that all three methods result in similar findings, though topic modeling produced a larger number of topics. For example, both the community detection and the cluster analysis identified the brands of HIV pre-exposure prophylaxis in one group, compared to separate topics in the topic analysis.

The community detection methods for the semantic networks provided very broad categories. This resulted in only two or three communities in each network that were interrelated. There are several advantages to organizing the textual data into succinct themes, including its simplicity and ability to describe the most prominent issues. Despite these advantages, there are still some nuances or details that may be missing when using the community detection method. For example, in the childhood vaccine semantic network, we find that the word *MMR* was included in the primarily HPV vaccine-related community. Further investigation would be needed to understand the primary concerns or discussions about the MMR vaccine in this dataset.

Topic modeling provided the most detailed method for examining the primary topics within the dataset. For example, the topic modeling specifically teased out different barriers against PrEP use, including separate topics for costs/pricing, access, patent issues, and potential side effects. In addition, the topic modeling elicited other topics that could have changed our perceptions of the issues related to PrEP. In particular, the topic on opinions related to Truvada commercials. The topic modeling results on PrEP barriers were similar to a manual content analysis on tweets related to PrEP (Calabrese et al., 2022). However, when considering the intertopic distance map, the number of specific topics is reduced and becomes more in line with the results from the community detection and cluster analysis.

Of course, topic modeling may not be the best fit for examining all datasets due to a lack of succinct topics and the potential for topics to overlap. While our diagnostic analyses indicated the ideal number of topics for the childhood vaccine dataset, many of the topics either involved several different issues or were more difficult to interpret. For example, Topic 3 focused on information related to *Gardasil* and the HPV vaccine generally, but also included information about the protests against the HPV vaccine in Ireland.

In addition, topic modeling required many more steps to determine the ideal number of topics and run the models when compared to the other methods for textual analyses. We ran many iterations to determine the ideal number of topics based on coherence, residuals, and other diagnostic scores when using topic modeling. While the results were much clearer, especially for the PrEP dataset, this may be an important factor when attempting to analyze large amounts of data in a short amount of time.

The cluster analysis provided the least detailed results from the three text analyses methods. This may largely be due to the disparity between word frequencies and co-occurrences. For example, the most common word in the PrEP dataset *Truvada* was mentioned 10,857 times, while the 30th most common word *health* was mentioned only 686 times. This resulted in many words that formed their own individual “cluster.” Despite this issue, cluster analysis still allowed us to organize the data, indicating the hierarchy of terms within each cluster. For example, the next level within the larger cluster in the PrEP data focused on barriers to PrEP, followed by eligibility, then the medication's function. Similarly, for the childhood vaccine dataset, the larger cluster included cervical cancer as the next level of prominence, followed by who can receive vaccination and the safety of the vaccines. This hierarchy of words allows us to better picture the most important aspects or concerns in the dataset.

This study is not without limitations. Our analysis relied on Twitter data, which may indicate why some of our methods resulted in less clear interpretations than others. Twitter users are limited to 240 characters in their posts and often use hashtags to increase the visibility of their posts. This may explain why our results had large differences in word frequencies. Future research should be conducted to determine the ability of these methods to deal with textual data from a variety of online and social media sources, as well as other texts. This will allow us to carefully depict which method is ideal for specific types of data sources. In addition, future work should examine managing textual data of different sizes and over time. How well do these analyses deal with the changing nature of text (Jiang et al., 2016; Calabrese et al., 2020)? Also, future work should examine how these analysis methods can handle languages other than English with different syntax, such as Korean and Chinese (Kwon et al., 2009; Ji et al., 2022). Lastly, the three methods we use in this study focus on the co-occurrence of words; however, advanced language models, such as ChatGPT (OpenAI, 2022) are now being developed to analyze text data through multiple techniques. For example, supervised machine learning may provide accurate outputs based on training datasets. While future work may examine these new, sophisticated text modeling methods, we should not count out our co-occurrence-based analyses. The methods used in our study have demonstrated the ability to examine textual data in meaningful yet succinct ways.

Overall, we find that all three text analysis methods (topic modeling, community detection in semantic networks, and cluster analysis) provide similar results to describe the subject matter of textual data. The topic modeling analysis was the most informative and produced the greatest number of topics related to the datasets. However, topic modeling may also lead to overlapping topics, which may be redundant or harder to interpret in some cases. The community detection and cluster analyses provide an overall broad view of the main themes related to the datasets but may fail to detect specific issues that topic modeling can potentially tease out. Our findings indicate that the ideal method for analyzing textual data may depend on the main goals of the researcher. Nevertheless, all three methods provided useful insight into the primary issues related to each dataset.

## Data availability statement

The original contributions presented in the study are included in the article/supplementary material, further inquiries can be directed to the corresponding author.

## Ethics statement

Ethical approval was not required for the study involving human data in accordance with the local legislation and institutional requirements. The data was accessed via Twitter's Premium API.

## Author contributions

GB conceptualized the study with JR. CC ran the method comparison and completed the initial draft of the manuscript. All authors contributed to the article and approved the submitted version.
